# Effect of Chromosomal Localization of NGS-Based Markers on Their Applicability for Analyzing Genetic Variation and Population Structure of Hexaploid Triticale

**DOI:** 10.3390/ijms25179568

**Published:** 2024-09-03

**Authors:** Justyna Leśniowska-Nowak, Piotr T. Bednarek, Karolina Czapla, Michał Nowak, Agnieszka Niedziela

**Affiliations:** 1 Institute of Plant Genetics, Breeding and Biotechnology, Faculty of Agrobioengineering, University of Life Sciences in Lublin, Akademicka St. 15, 20-950 Lublin, Poland; justyna.lesniowska@up.lublin.pl; 2 Plant Breeding and Acclimatization Institute—National Research Institute, Radzików, 05-870 Błonie, Poland; p.bednarek@ihar.edu.pl; 3 Department of Biochemistry and Molecular Biology, Faculty of Medical Sciences, Medical University of Lublin, Chodźki St. 1, 20-093 Lublin, Poland; karolina.czapla@umlub.pl

**Keywords:** triticale, genetic variation, population structure, DArTseq

## Abstract

This study aimed to determine whether using DNA-based markers assigned to individual chromosomes would detect the genetic structures of 446 winter triticale forms originating from two breeding companies more effectively than using the entire pool of markers. After filtering for quality control parameters, 6380 codominant single nucleotide polymorphisms (SNPs) markers and 17,490 dominant diversity array technology (silicoDArT) markers were considered for analysis. The mean polymorphic information content (PIC) values varied depending on the chromosomes and ranged from 0.30 (2R) to 0.43 (7A) for the SNPs and from 0.28 (2A) to 0.35 (6R) for the silicoDArTs. The highest correlation of genetic distance (GD) matrices based on SNP markers was observed among the 5B–5R (0.642), 5B–7B (0.626), and 5A–5R (0.605) chromosomes. When silicoDArTs were used for the analysis, the strongest correlations were found between 5B–5R (0.732) and 2B–5B (0.718). A Bayesian analysis showed that SNPs (total marker pool) allowed for the identification of a more complex structure (K = 4, ΔK = 2460.2) than the analysis based on silicoDArTs (K = 2, ΔK = 128). Triticale lines formed into groups, ranging from two (most of the chromosomes) to four (7A) groups depending on the analyzed chromosome when SNP markers were used for analysis. Linkage disequilibrium (LD) varied among individual chromosomes, ranging from 0.031 for 1A to 0.228 for 7R.

## 1. Introduction

Combining wheat and rye to create a new species was first proposed in the 19th century [1]. However, most breeding efforts started in the early 20th century [2]. Triticale combined rye’s resistance to rye-specific diseases and pests with wheat’s productivity and quality characteristics [3]. The key advantages of hexaploid triticale (AABBRR; 2n = 6x = 42) result from a cross between *Triticum turgidum* subsp. durum (2n = 4x = 28; AABB) and *Secale cereale* L. (2n = 2x = 14; RR), and include robust root systems [4], low soil requirements [5], drought tolerance [6], and disease resistance [7]. Hexaploid triticale is a vital forage crop and a promising energy crop [8,9].

The classification system based on how genotypes are obtained divides triticale into the categories of primary and secondary. Primary triticale genotypes are hybrids obtained by crossing rye with wheat, whereas secondary genotypes are obtained via crosses among primary triticale by crossing primary triticale lines with secondary triticale lines or primary triticale’s with wheat or rye [10]. Currently, secondary triticale forms (a cross between two types of primary (first-cross) triticale’s), formed as a result of the combination of the chromosomes from genomes A and B, which increased genetic variation, fulfil breeders’ needs [11,12,13]. Due to its origin, triticale is expected to show narrow variation and a weak structure within species. Thus, studying genetic variation is a prerequisite for future perspectives and the successes of breeding programs.

Using factorial analysis, over one thousand hexaploid winter triticale genotypes from 24 countries were subjected to morphological and physiological assessments of their yield components. The results revealed that the first three principal components may account for up to 80% of the variance [14]. Several researchers have conducted similar experiments [11,15]. However, given the limited number of traits that can be researched, the majority of which are quantitative, and their reliance on environmental factors, such a study is only tangentially significant [16]. The introduction of molecular markers has enabled the refinement of this kind of study. To determine if 80 different European hexaploid triticale lines had a genetic structure, Kuleung et al. [17] used 57 simple sequence repeat (SSR) markers. There was a genetic variance ranging from 7% to 86%. According to an agglomeration analysis, the materials formed five groups, which generally corresponded with a pedigree. This clustering may result from the fact that breeders also use the varieties of other breeders as parents in their breeding programs, which reduces the genetic differentiation between their breeding programs. Researchers looked at 232 types of triticale from Polish breeding companies and genotyped them with diversity array technology (DArT) markers. They found that the genetic structure of the material was weak, with 86% of the variation being found within groups [18]. Similar outcomes were found for 161 lines from the global collection [19]. For DArT markers, a principal coordinates analysis (PCoA) displayed just two data groupings. The first two coordinates explained 23.4% of the variation. Additionally, 144 triticale forms were evaluated using DArT markers, revealing a genetic identity of 60% [20]. Surprisingly, an analysis of 54 Brazilian genotypes from two breeding organizations employing SSR markers revealed as many as seven groups [21].

In the case of triticale, clustering usually differentiates between the winter and spring varieties. The spring forms show slightly more variance than the winter forms [19,20]. Other crops, such as wheat [22,23,24], barley [25], and rapeseed, have also shown this distinction between their winter and spring varieties [26]. The population’s geographic origin is another element that typically has the most significant impact on population clustering [27,28]. It must be emphasized that grouping triticale materials into separate groups may be difficult, regardless of the marker system used for analysis. For instance, Losert et al. [29] examined 121 registered triticale varieties using genotyping by sequencing (GBS) markers. No genetic structure related to the known origin was discovered. Tams et al. [30] revealed the same phenomenon for 128 genotypes of winter European triticale using SSRs. The PCoA’s first two coordinates accounted for 22.4% of the variation in materials from other nations. Studies utilizing other molecular techniques do not always support the assumption that triticale has a slight genetic variation and a tiny structure [18]. Yet, most triticale population assessments indicate either a few groups or none [18,29,31]. It depends on how many genetic variants are in the gene pool, how much genome-wide linkage disequilibrium (LD) there is, and how closely related the population is to itself. These factors affect the mapping resolution, marker density, statistical methods, mapping power, and how well plant breeding works [32].

According to recent research, triticale agronomic traits should be examined separately for each chromosome rather than for all simultaneously [33]. Rye provided evidence of selection resulting from years of breeding at the chromosomal level [34]. The different breeding preferences among plant breeding companies in the same country support this evidence. When variation is examined at the level of a species’ chromosomes, it can be used to analyze the genetic structure of the species. Also, the genetic structure found when counting the total number of a specific type of genetic marker might be different from the genetic structure found by counting the markers attached to specific chromosomes. Moreover, the genetic structure may vary depending on the DNA marker type (dominant vs. codominant).

The goal of our study is to determine whether DNA markers attached to specific triticale chromosomes would work better than using all markers together to determine the genetic structures of triticale types originating from two breeding companies.

## 2. Results

The initial data set consisted of 24,353 SNPs and 64,334 silicoDArTs. After filtering for quality control parameters, 6380 SNPs and 17,490 silicoDArT markers were considered for analysis. The number of mapped SNPs per chromosome ranged from 93 (4B) to 416 (6R), whereas the number of silicoDArTs varied from 180 (4B) to 946 (4R) (Figure S1). In general, the R genome showed the highest marker number. As many as 1740 SNPs (27%) and 6192 silicoDArTs (35%) were not assigned to chromosomes but were still used for the analysis based on the total pool of markers.

**Figure 1:**
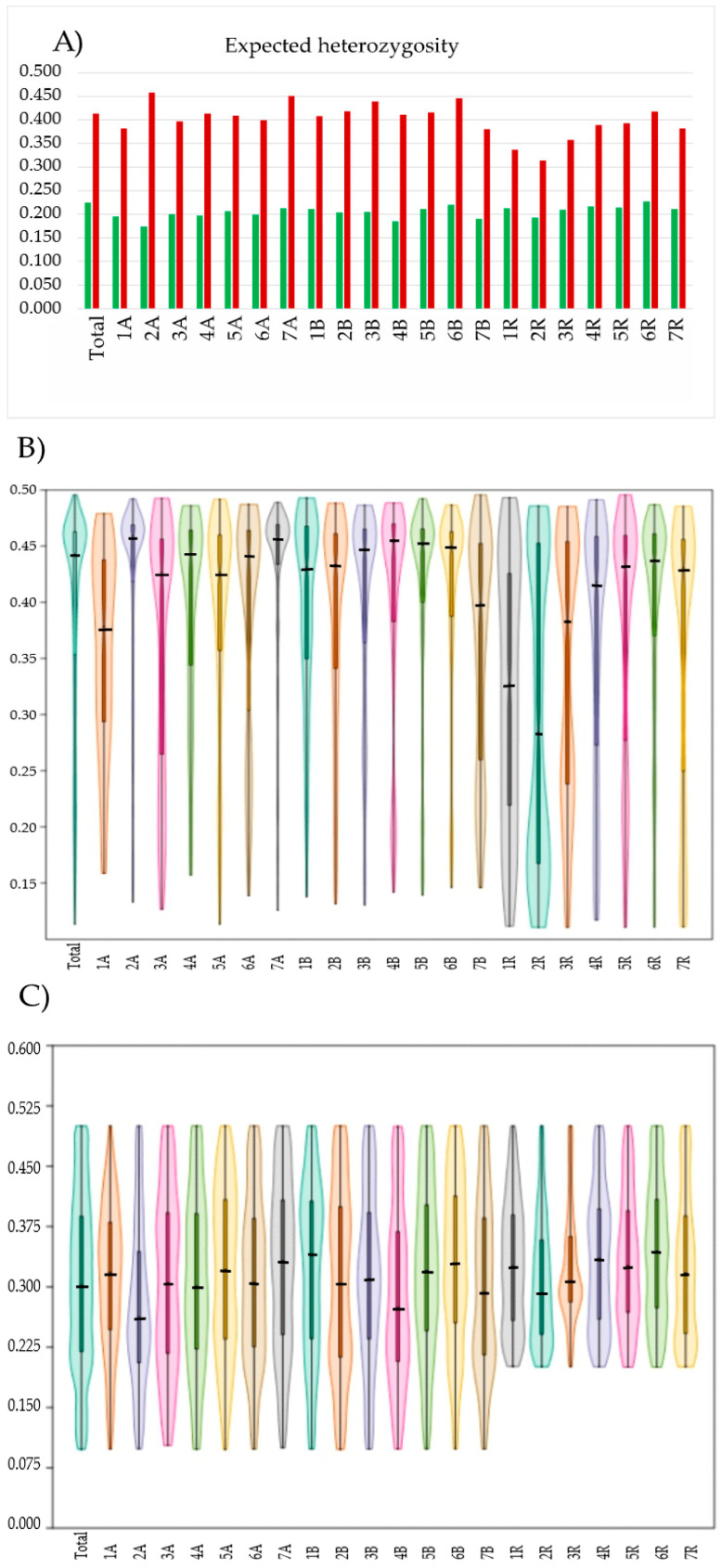
Average values of expected heterozygosity (*H_e_*) (**A**) and distribution of the polymorphic information content (PIC) values of SNP (**B**) and silicoDArT (**C**) markers calculated for the total marker pools and particular chromosomes of triticale. The horizontal line inside the box shows the median. The shapes of violin plots show the distribution of PIC values by chromosome.

A marker’s polymorphic information content (PIC) delivers relevant information on its usefulness for studies using the given material set. The higher the value, the more informative the marker [35], with a value of about 0.5 considered to be the most informative. In our study, the PIC values for both marker types ranged from 0.1 to 0.5, with the mean values for the total marker pools being equal to 0.39 and 0.30 for SNPs and silicoDArTs, respectively. The proportions of markers with a PIC value above 0.41 (highly informative) reached 64.9% (SNP) and 20.5% (silicoDArTs). The mean PIC values for markers with a chromosome location varied from 0.30 for 2R to 0.43 for 7A (SNPs) and 0.28 for 2A to 0.35 for 6R (silicoDArTs). The SNP markers from each chromosome had similar PIC values, with most markers being above 0.41 (48% for 7B and 77% for 2A). Except for the 1A and 1R chromosomes, which had a lot of markers with PIC values below 0.40 (59% for 1A and 68% for 1R), none of the other chromosomes did. However, the differences between triticale genomes were significant (F = 6.534, *p* < 0.05). Tukey’s HSD test showed that the R genome formed a separate group. The PIC distribution of silicoDArTs indicated the highest proportion of markers (ca. 41% (4A and 7B) to 74% (3R)), with values between 0.25 and 0.40, except for 2A and 4B, where the highest proportion of markers (ca. 38%) was noticed in the range from 0.1 to 0.25, referring to markers with a medium informativeness. The difference between triticale genomes was significant (F = 4.74, *p* < 0.05). Tukey’s HSD test showed that the A and R genomes were in separate groups. Violin plots showing the distribution of PIC values by chromosome are given in Figure 1.

Expected heterozygosity (*H_e_*) is a common statistic for assessing the amount of genetic variability within a population. The *H_e_* values for the total SNP markers pool ranged from 0.12 (4 SNPs) to 0.50 (912 SNPs), with an average of 0.41 (Figure 1). The average *H_e_* value noticed for the markers assigned to each chromosome was the highest for 2A (0.46) and the lowest for 2R (0.31). The differences between triticale genomes were significant (F = 5.902, *p* < 0.05). Tukey’s HSD test showed that the R genome formed a separate group. When silicoDArTs were applied for analysis, the *H_e_* for individual markers varied from 0.05 (363 markers) to 0.50 (180 markers), with an average of 0.22. The parameter depended on chromosomes and was the highest for 6R (0.23) and the lowest for 2A (0.17). According to AMOVA, the difference between the triticale genomes was insignificant (F = 2.683, *p* = 0.09). However, the average *H_e_* for the A, B, and R genomes within the given marker tape ranged from 0.393 (A) to 0.396 (B) and from 0.308 (A) to 0.329 (R) in the case of SNPs and silicoDArTs, respectively.

Based on the total pools of SNP and silicoDArT markers, the UPGMA clustering of plant materials using Nei’s genetic distance (Figure S2A,C) showed that the two main groups differed slightly depending on the marker system used. The first cluster constructed using SNP markers included 119 out of the 254 genotypes from Strzelce Plant Breeding and 164 among the 192 lines from DANKO Plant Breeding. The second cluster splintered into two subclusters. The first subcluster included 70 triticale lines from Strzelce and four lines from DANKO, whereas the second subcluster comprised 65 and 24 lines from Strzelce and DANKO, respectively. Based on silicoDArTs, cluster one included 58 lines (46 from Strzelce and 12 from DANKO). Cluster two consisted of the remaining 388 lines, further divided into two branches: 113 lines from Strzelce and 34 lines from DANKO formed one branch, and 95 (from Strzelce) and 146 (from DANKO) lines formed another.

The highest Nei’s genetic distance (GD) calculated using the total pool of SNP markers was between the L202 line and L80 or L135 (>11,000), and the lowest (<1000) was between B4 and B80, B261 and B262, L5 and L35, L27 and L28, L31 and L129, and L36 and L125. Similarly, the L202, B4, and B27 lines had the highest GDs (>10,000), with 28, 28, and 36 lines among the investigated pool. When silicoDArTs were utilized, the highest GD values (>8500) were observed between B39 vs. B120 and B143 and B115 vs. B146 and B192. Lines B120 and B143 showed the highest GD values (>8000), with 16 and 17 lines, respectively. The lowest values (<300) occurred between L31 and L129, L36 and L125, L4 and L200, L6 and L171, L27 and L28, L14 and L201, and B261 and B262.

The first two principal axes in the PCoA utilizing the whole pool of SNPs explained only 5.4% and 5.0% of the total genetic variation, respectively. The total pool of silicoDArTs resulted in comparable values (6.6% and 4.1% for the first and second principal coordinates, respectively). However, separate accession groups could not be seen independently of the marker system applied for analysis (Figure S1B,D). Still, in the case of silicoDArTs, materials from DANKO showed less variation than those from Strzelce. However, a comparable dispersion was seen for all analyzed forms when applying the SNP markers.

Mantel tests used to show the correlation level between two distance matrices, reflecting how the lines differed from each other at the genetic level, revealed a significant correlation between two genetic distance matrices based on the total pool of SNPs and silicoDArTs (r = 0.505; *p* = 0.01). The correlation between distance matrices representing the same chromosome but evaluated on SNPs and silicoDArTs indicated a significant correlation that ranged from 0.074 to 0.387 for 4R and 3R, respectively. Correlation coefficients were sometimes insignificant (1A, 3A, 4A, 1B, and 5R) (Table 1).

Mantel tests showed that the GD matrices evaluated on the markers assigned to triticale chromosomes were positively correlated. The correlation was relatively high between 5B and 5R (0.642), 5B and 7B (0.626), and 5A and 5R (0.605) when SNP markers were considered (Table 2). The 4B- and 6B-based GD matrices exhibited the lowest correlation values (0.175–0.321) of all the matrices, including those derived from a whole set of markers. When silicoDArTs were used for analysis, the highest correlation was observed between 5B and 5R (0.732), 2B and 5B (0.718), 1B and 1R (0.711), 1B and 2B (0.709), 1B and 5B (0.700), 5A and 5B (0.704), and 5A and 5R (0.700). The lowest values noticed for 3R-based matrices were correlated with all remaining matrices, excluding those for 3B.

**Figure 2:**
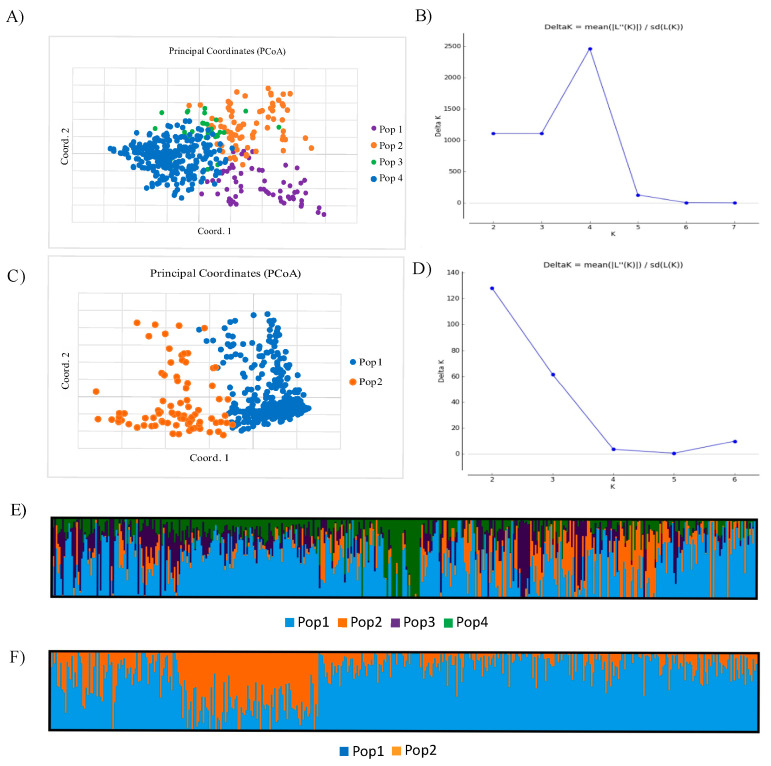
Principal coordinate analysis (PCoA) based on Nei’s genetic distance inferred from the (**A**) SNP and (**B**) silicoDArT data of a 446 triticale accessions. Each of the color of dots indicate individuals assigned to different subpopulations (Pop). Delta K for differing numbers of subpopulations (k) based on: (**C**) SNP and (**D**) silicoDArT markers. Structure plot with K = 4 and K = 2 clusters of triticale accessions based on: (**E**) SNP and (**F**) silicoDArT markers. Each accession’s genome is represented by a single row, which is partitioned into colored segments in proportion to the estimated membership in the subpopulations.

A Bayesian analysis of the genetic structure based on SNP markers revealed that the highest ΔK value was 2460.2 (K = 4), indicating the presence of four groups (Figure 2A,C). The respective groups encompassed 65, 75, 31, and 275 accession numbers and formed Pop1, Pop2, Pop3, and Pop4, respectively (Figure 2A,E). The origin of the accession was not a determinant of the genetic structure. The lines originating from DANKO and Strzelce were present in each group, but in different proportions. The percentages of lines from DANKO in individual populations were 37.0% in Pop1, 76.0% in Pop2, 16.1% in Pop3, and 38.5% in Pop4. Different results were obtained when the total pool of silicoDArT markers was applied. A sharp peak at K = 2 was identified, but ΔK reached only 128.0, indicating a weak population structure (Figure 2D). Among the investigated lines, 363 clustered in Pop1 and 83 clustered in Pop2 (Figure 2B,F). The clustering did not follow known genotype origins. However, only two genotypes from DANKO were included in Pop2.

Codominant SNP markers, considered to be more informative, more strongly illustrated the population structure in our study compared to dominant silicoDArTs, and were used to analyze population structures based on markers assigned to individual chromosomes. The results preferentially indicated the presence of two groupings which formed Pop1 and Pop2 (2A, 3A, 6A, 7A, 1B, 4B, 5B, 6B, 7B, 1R, 4R, 5R, 6R, and 7R) (Table 3). However, for chromosomes 1A, 5A, 2B, 3B, 2R, and 3R, the highest ΔK value was found to be the greatest for K = 3, and the highest K = 4 was evaluated for the 4A chromosome (Table 3). The triticale genotypes assigned to the particular groupings differed depending on chromosome, even if they had the same K value.

An Analysis of Molecular Variance (AMOVA) revealed that 14% of the total variance (*Phi_PT_* = 0.139, *p* < 0.001) was found among four subpopulations estimated from STRUCTURE when the entire pool of SNPs was used (Table 3). Different results were found depending on the chromosome. The highest values between population variance accounted for 41% (*Phi_PT_* = 0.413, *p* < 0.001) for the 7R chromosome, whereas low values (i.e., *Phi_PT_* = 1%, *p* < 0.001) were evaluated for 4R and 7B, respectively. The *Phi_PT_* between particular populations with K > 2 (chromosomes 1A, 4A, 5A, 2B, 3B, 2R, and 3R) was highly significant (*p* < 0.001) and varied from 0.015 (between Pop2 and Pop3 for chromosome 3R) to 0.351 (between Pop2 and Pop3 for chromosome 2R) (Table 4). High values of *Phi_PT_* (greater than 0.20) obtained for 2A, 7A, 1B, 2B, 4B, 6B, 1R, 2R, 5R, 6R, and 7R were indicative of a high genetic differentiation between grouping accessions.

**Figure 3:**
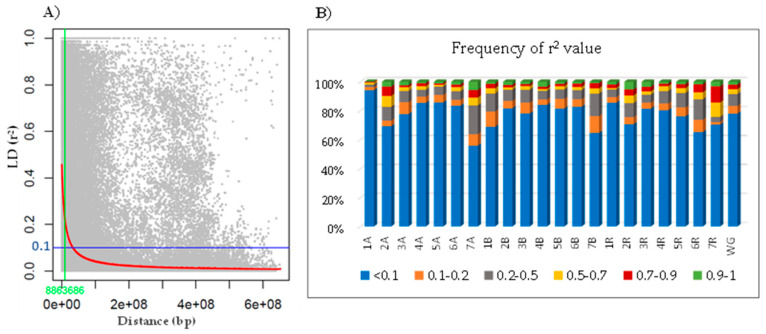
Linkage disequilibrium (LD) decay plot for the whole genome of triticale lines and frequency of *r*^2^ values. (**A**) Scatter plot of LD values of intra-chromosomal pairwise loci against physical distance (Mb). LD decay was fitted with the nonlinear regression model curve (red line). The green line indicates the intersection between critical *r*^2^ and the map distance. The critical threshold of *r*^2^ value below which the LD could be considered caused by physical linkage was set to 0.1 (blue line). (**B**) LD (*r*^2^) values frequency by chromosome and whole genome (WG).

An analysis of linkage disequilibrium (LD) for the whole genome showed that 36% of the SNP marker pairs were associated with a *p*-value below 0.001, 78% of which recorded an *r*^2^ value of less than 0.1 (Table 5). The mean value of *r*^2^ was 0.122. Pairs of loci in significant LD on the chromosome level varied from 11.6% (1A) to 55.2% (7A), and *r*^2^ values of <0.1 ranged from 55.7% (7A) to 94.1% (1A). The highest (*r*^2^ > 0.7) or complete (*r*^2^ = 1.0) LD was represented in only 0.57% of the significant pairwise comparisons for chromosome 1A, but reached approximately 10% for 7A, 2A. and 2R and exceeded 14% for chromosome 7R. The distribution of *r*^2^ values is shown in Figure 3.

The mean LD varied among individual chromosomes and ranged from 0.031 for 1A to 0.228 for 7R (Table 5). The average LD decay for the whole genome was 8.86 Mb when *r*^2^ = 0.1 (Figure 3). The LD decay for a predicted *r*^2^ of 0.1 varied from 1.18 Mb (1A) to 28.36 Mb (7R) based on the chromosomes. The mean distance between mapped SNP markers ranged from 2.53 Mb for chromosome 6R to 7.32 Mb for chromosome 7B and was 4.24 Mb for the whole genome (Table 5).

## 3. Discussion

The decision on the marker type suitable for a given task could be more straightforward. Dominant or codominant markers are available on one side, but rarely both. On the other hand, the choice is nearly always taken a priori. Thus, it is vital to have reasonable criteria to facilitate this decision. The DArT PL company offers a DArTseq platform generating codominant (SNP) and dominant (silicoDArT) markers based on a single data set. In this study, we highlighted the efficiency of both marker types for genetic diversity analyses in the entire triticale collection. In addition, a population structure and linkage disequilibrium (LD) study was also conducted using a whole pool of SNPs and SNPs assigned to individual chromosomes.

The PIC values evaluated in the study based on triticale lines showed that SNPs seemed more informative than silicoDArTs. PIC values equal to 0.39 for SNP markers are congruent with data presented for the other plant species, such as rye [36], *Pisum fulvum* [37], and maize [38]. The results indicated that the marker system and marker types were informative enough for our purpose, which was also the case at the level of genomes, with some preferences for the marker type used. The results were more uniform for silicoDArTs, with a mean PIC equal to 0.31 for the A and B genomes and 0.33 for R the genome. Comparing the genomes based on the PICs evaluated on the SNPs showed that the R genome (mean PIC = 0.35) separated from the wheat genome (mean PIC = 0.40 for the A and B genomes). Within individual genomes, the PIC varied slightly between chromosomes and ranged from 0.28 (2A) to 0.33 (7A), from 0.29 (4B) to 0.33 (6B), and from 0.31 (2R) to 0.35 (6R) for dominant silicoDArT markers. The PICs within individual genomes for codominant SNP markers reached maximum values of 0.43, 0.41, and 0.40 for 7A, 5B, and 6R, respectively. The mean value of the expected heterozygosity calculated by SNPs was twofold higher (*H_e_* = 0.41) than that for silicoDArTs (*H_e_* = 0.22), most probably due to the codominant nature of SNP markers. Similar relationships were observed, regardless of whether they were calculated using whole marker pools or markers assigned to individual chromosomes. However, for SNP markers, the mean *H_e_* values were lower for the R genome than for the A and B genomes. The *H_e_* index value for the triticale collection was much higher than that for breeding lines of common wheat [39] or barley [40], and it was about the same as that for a group of wheat genotypes with a lot of genetic diversity [41]. The differences we saw between the PIC and *H_e_* in this study were due to marker types that showed their dominant and codominant natures. On the other hand, differences between genomes are likely related to how materials are bred by their companies. Thus, the PIC and *H_e_* indices could be used to decide which marker system better fits the requirements of given studies. To better discriminate between lines, preferences for higher PIC values and lower *H_e_* values should be given. Furthermore, the presented data indicate that each chromosome, at least in some cases, could be treated individually for a better resolution.

UPGMA analyses showed that data clustering utilizing either silicoDArTs or SNPs did not group plant materials based on their breeding origin. Materials from the two companies were evenly distributed between two main clusters utilizing SNPs and silicoDArTs. A grouping like this may mean that companies either share breeding materials or that the species’ genetic pool is limited. An alternative explanation is that companies’ breeding preferences are similar, or breeding pressure needs to be stronger or longer to form separate genetic pools representative of such companies. Moreover, it cannot be excluded that this pressure is directed preferentially towards specific chromosomes, as only a minor fraction of agronomic traits is considered in breeding programs. If so, whole marker pools may not identify such differences. While a limited genetic pool in triticale is apparent [18] and the exchange of breeding materials cannot be excluded, we tend to think that, at least to some extent, breeding companies exhibit distinct pressure towards their materials. Still, time is needed to evaluate the differences between pools. Thus, these differences are subtle or averaged by the whole marker pool representing all chromosomes simultaneously, indicating that putative distinction could be uncovered based on markers assigned to selected chromosomes. Nevertheless, the outcome from the PCoA analysis based on the whole pools of markers was that the materials from DANKO Plant Breeding Ltd., located in Laski, Poland, were more uniform than those from Strzelce Plant Breeding Ltd., located in Borowo, Poland, as indicated by clustering using silicoDArTs. Furthermore, both total pools of SNPs and silicoDArTs were inconclusive regarding the differentiation of plant materials due to their origin, indicating that the marker choice may have led to somewhat distinct and inconsistent results. This conclusion is congruent with the results presented by other studies [34,42,43] and is supported by the correlation between genetic distance matrices evaluated based on SNPs and silicoDArTs. This result encouraged us to test whether using markers associated with individual chromosomes would be more conclusive for plant material differentiation. We chose to compare genetic distance matrices evaluated based on SNP and silicoDArT markers assigned to triticale chromosomes for that purpose.

Interestingly, the correlations varied even more than in the total marker pools. Moreover, different results were obtained depending on the marker system used. Within each type of marker, the correlation values between chromosomes ranged from low but still significant to fairly high. This may suggest that the most correlated chromosomes were under the same breeding selection, exhibited synteny, or carried some agronomically important traits important for breeding programs. The correlation between 5A and 5R partly supports the notion concerning directed breeding pressure or synteny regarding agronomic traits. Both chromosomes code QTLs conferring height with the dwarfing gene *Ddw1*, which is localized on 5R in rye [44], and the dominant height-reducing *Rht12* locus, located on 5A in wheat [45]—both QTLs control plant height in central European winter triticale [46]. In addition, many other vital QTLs responsible for powdery mildew resistance, seedling freezing tolerance, and yielding capacity have been localized in triticale on 5R or 5A [47,48,49,50].

Moreover, the homology between 5RS and 5AS and between 5RS and 5BS (the most correlated GDs) has been proven by many researchers [51,52,53] and is congruent with our Mantel test results. Another example is the 1B and 2B pair. Here, the two chromosomes harbor numerous R-genes in wheat and triticale, including functional stripe rust [54,55] and powdery mildew R-genes [56,57]. A high correlation coefficient between chromosomes from the same homologous group, e.g., 5A and 5B, confirms the presence of essential genes responsible for a plant’s development, adaptation, or resistance.

Regarding the presented data, it is reasonable to assume that different marker types (codominant SNPs or dominant silicoDArTs), combined with Bayesian statistics, should detect the distinct genetic structures of the analyzed triticale population. However, only the SNP markers (total marker pool) supported the strong stratification of triticale materials (ΔK = 2460.2), with moderate differentiation between Pops (*Phi_PT_* ranging from 0.150 to 0.237). Thus, we focused on codominant SNP markers in later structure and LD analyses. The Bayesian-model-based method based on markers assigned to individual chromosomes resulted in identifying distinct population structures. The differentiation was more apparent compared to the total marker pools, respectively. Since there was no information on breeding preferences, physical traits, etc., it was hard to determine what the genetic structures found meant.

LD is an essential parameter of genetic diversity in natural populations and germplasm collections, and it can be employed to evaluate population stability and crop improvement programs [58]. The extent and distribution of LD in the genome determine the required number of SNP markers and the mapping resolution for the success of forward genetic studies, such as genome-wide association analyses [59]. However, in cases of triticale, when SNPs’ positions are based on the genomes of single genotypes of rye and wheat, we cannot exclude spurious results in LD analysis [60]. It has been reported that 10–20% of SNPs in plant species may need to correct their physical positions due to the available reference assembly [60,61].

In our study, the number of SNP marker pairs with significant LD (*p* < 0.01) varied from 11.6% to 55.15%, depending on the chromosome analyzed. The mean LD was the lowest for the 1A (*r*^2^ = 0.031) and the highest for the 7R (*r*^2^ = 0.228) chromosomes. This result was expected and is consistent with similar analyses in bread wheat [62], durum wheat [63], and rye [64]. However, much higher LD values exceeding 0.300 and reaching ~0.420 for the 3R and 4R chromosomes were reported by Vendelbo et al. [64] for non-restorer germplasm and cytoplasmic male-sterile lines of rye, who explained that the higher LD in the investigated population was linked to the low frequency of detectable recombination events caused by the depletion of rare alleles and suggesting the occurrence of population bottlenecks or intense selection. The same explanation was suggested by Roncallo et al. [63] for the rising number of SNPs in high (*r*^2^ > 0.07) or complete LD (*r*^2^ = 1.0) for the 2A and 7A chromosomes in a durum wheat collection, compatible with our results observed for the identical chromosomes, as well as for 2R and 7R. In our study, the maximum LD decay distance (*r*^2^ > 0.1) was 28.4 Mb, with an average LD decay distance of about 8.9 Mb for the triticale genome. This overall LD decay distance is difficult to compare with other studies due to the different methods used in calculations [65], as well as the use of recombination units (cMs) instead of base pairs (bps). Moreover, self-fertilizing plants usually show less decay of LD because, in a homozygous genetic background, recombination events are ineffective in causing LD decay [59]. However, the slightly open-pollinating nature of triticale, which can be up to 10% [66], may influence this fact.

The presented results demonstrate that analyses of any genetic pool should be supported by selecting the most adequate marker system, which may vary depending on the genome or individual chromosome levels. The more informative the system is, the better the available outcomes. Moreover, depending on specific requirements, it might be vital to base a study on more heterozygotic or homozygous materials. When it is crucial to identify the most exhibited differences based on genetic distances, the more uniform the analyzed materials are, the better. Such data are significant when, i.e., choosing crossing lines when the maximum heterozygosity is expected in the F1 progeny to compensate for the adverse effects of some recessive alleles present in the homozygous materials. As any breeding materials can be subjected to varying breeding programs, the results could be addressed exclusively to the given species under the assumption that the representative genetic pool is analyzed. Furthermore, similar studies conducted at the level of individual chromosomes are time-consuming and hardly available, which makes conclusions concerning other cereals difficult.

## 4. Materials and Methods

### 4.1. Plant Material

The plant material consisted of 446 homogeneous breeding lines of winter hexaploid triticale (X Triticosecale Wittm. ex A. Camus) obtained from the Polish plant breeding companies DANKO Plant Breeding Ltd., located in Laski (51°48′14″ N, 21°8′41″ E) (192 genotypes), and Strzelce Plant Breeding Ltd., IHAR Group, located in Borowo (52°7′4″ N, 16°46′53″ E) (254 genotypes). The breeding lines for examination were selected by the breeders to represent the genetic variability of triticale within each company’s breeding materials to the maximum possible extent.

### 4.2. DNA Extraction and Genotyping

The frozen leaf samples collected from triticale at the heading stage of development were homogenized in liquid nitrogen using a mortar and pestle. The homogenate was transferred to 2 mL Eppendorf tubes, and then the total DNA was isolated using a commercial reagent kit, the DNeasy Plant Mini Kit (Qiagen, Hilden, Germany). The isolated DNA samples were qualitatively and quantitatively evaluated using a NanoDrop 2000 spectrophotometer (Thermo Fisher Scientific Inc., Waltham, MA, USA). DNA integrity was tested electrophoretically in 1.5% agarose gel, and 100 ng/mL samples were prepared. These samples underwent sequencing (Diversity Arrays Technology Pty Ltd., Canberra, Australia; http://www.diversityarrays.com/ (accessed on 1 June 2024)) at a 2.5 mln depth of reads using the HiSeq 2000 platform (Illumina Inc., San Diego, CA, USA). Two marker types were generated by DArTseq analysis: SNPs (codominant) and silicoDArTs (dominant). The codominant markers were scored as 0, 1, and 2, where 0 and 1 were homozygotes. The dominant markers were coded as 0 and 1. The chromosomal assignment and position of the markers were evaluated based on wheat (Wheat Chinese Spring IWGSC RefSeq v1.0) and rye (Secale cereale Lo7 RefSeq v3.0; https://galaxy-web.ipk-gatersleben.de/ (accessed on 1 June 2024)) sequences in reference genomes that were homologous to the marker sequence. Diversity Arrays Technology Pty Ltd. supported the data.

### 4.3. Quality Control of Markers and Genetic Diversity

Marker quality was based on the following parameters: reproducibility > 95%, call rate > 95%, minor allele frequency (MAF > 0.05), and one ratio > 0.1 [67]. The scoring of reproducibility involved the proportion of technical replicate assay pairs for which the marker score exhibited consistency. The call rate determined the proportion of genotypes per marker with non-missing data. Finally, the one ratio represented the proportion of samples for which the genotype score equaled ‘1’. The quality parameters and polymorphic information content (PIC) of each marker were computed automatically by the DArT software (version 3.5.2).

The expected heterozygosity (*H_e_*) of the total SNP and silicoDArT markers that met the filter criteria was calculated using GenAlEx v. 6.5 [68]. The expected heterozygosity (*H_e_*), also known as the gene diversity of the locus [69], is a measure of the genetic diversity in a population and describes the expected proportion of heterozygous genotypes if the population is in Hardy–Weinberg equilibrium. The *H_e_* index is calculated according to Nei [70].

### 4.4. Statistics

A one-way analysis of variance (ANOVA) was used to determine if there were significant differences in the mean values of *H_e_* and PIC for the triticale A, B, and R genomes. The AMOVA was evaluated in Statistica v.13.3.0 [71]. Violin plots visualized the distributions of PIC values among chromosomes and were constructed in PAST version 4.17 software [72]. An unweighted pair group method with arithmetic mean (UPGMA) and 1000 bootstrap replications using the PAST software [72] based on Nei’s genetic distance was conducted. Then, the genetic relationships among triticale lines, including their origins (DANKO Plant Breeding Ltd. and Strzelce Plant Breeding Ltd.), were assessed.

Nei’s genetic distance (GD) matrices [70] and principal coordinate analyses (PCoAs), as well as the Mantel test [73], were conducted in GenAlex v. 6.5 [68].

### 4.5. Population Structure

The population genetic structure was evaluated utilizing a Bayesian algorithm implemented in Structure software version 2.3.4 [74]. With correlated allele frequencies, the admixture model with 50,000 lengths of the burning period and 100,000 MCMC replicates was applied. Ten independent runs were performed for each simulated value of K, ranging from 1 to 7. The best K was identified based on the approach of Evanno et al. [75]. The structure results were visualized via the online server (http://clumpak.tau.ac.il/, (accessed on 1 June 2024)) in the CLUMPAK software (version 1.1.2) [76]. The “main pipeline-admixture” and “best K” algorithms were used. The analysis was conducted based on the total silicoDArT and SNP marker pools. In the case of SNP markers, the analysis was conducted on markers assigned to particular chromosomes.

An analysis of molecular variance (AMOVA) was performed based on the total pool of SNPs and SNPs assigned to individual chromosomes. Furthermore, the pairwise *Phi_PT_* population values were calculated to estimate the genetic differentiation among recognized populations (Pops). Finally, probability values estimated by 1000 permutations were used to determine whether the partitioning of variance components was significant. The genetic indices and AMOVA were evaluated in GeneAlEx v. 6.5 [68].

### 4.6. Linkage Disequilibrium

Linkage disequilibrium (LD) was calculated in the TASSEL 5.0 software [77] with a significance level of *p* < 0.001 and a sliding window of 50 SNPs. The chromosome and genome-specific mean values were estimated. LD (*r*^2^) was plotted against the physical distance (bp) between markers to determine the LD decay distance (bp). A trend line describing the LD decay was evaluated by a nonlinear regression model based on Hill and Weir [78] and implemented in Remington et al. [79] in R (http://www.rproject.org; (accessed on 1 June 2024)). The critical threshold of the *r*^2^ value, below which the LD could be considered as being caused by physical linkage, was set to 0.1.

## 5. Conclusions

This study concludes that both codominant (SNP) and dominant (silicoDArT) markers efficiently analyze triticale genetic diversity. However, SNP markers were found to be more informative than silicoDArTs. It is important to note that the choice of marker type can lead to distinct and inconsistent results. Additionally, the study showed correlations between the genetic distance matrices of specific chromosomes, indicating shared breeding selection or the presence of important agronomic traits. By using SNP markers in combination with Bayesian statistics, this study achieved a strong classification of triticale materials based on markers assigned to individual chromosomes, and such population structures differed depending on whether the analyses were conducted on individual chromosome-based markers or their total pool. An LD analysis based on SNPs revealed variations in the LD across different chromosomes, but the LD values were relatively low, allowing us to work on the whole marker pool and markers assigned to chromosomes.

## Figures and Tables

**Table 1:** Mantel test evaluated between genetic distance matrices of individual chromosomes calculated based on SNPs and silicoDArTs.

Chromosome	Correlation Coefficient (*r*)	Determination Coefficient (*r*^2^)	*p*
Total_SNP vs. Total_silicoDArT	0.505	0.255	0.01
1A_SNP vs. 1A_silicoDArT	0.027	0.0007	0.22
2A_SNP vs. 2A_silicoDArT	0.120	0.0144	0.01
3A_SNP vs. 3A_silicoDArT	0.036	0.0013	0.14
4A_SNP vs. 4A_silicoDArT	0.028	0.0008	0.22
5A_SNP vs. 5A_silicoDArT	0.109	0.0118	0.01
6A_SNP vs. 6A_silicoDArT	0.096	0.0093	0.01
7A_SNP vs. 7A_silicoDArT	0.169	0.0286	0.01
1B_SNP vs. 1B_silicoDArT	0.021	0.0004	0.15
2B_SNP vs. 2B_silicoDArT	0.238	0.0566	0.01
3B_SNP vs. 3B_silicoDArT	0.215	0.0463	0.01
4B_SNP vs. 4B_silicoDArT	0.110	0.0121	0.01
5B_SNP vs. 5B_silicoDArT	0.307	0.0943	0.01
6B_SNP vs. 6B_silicoDArT	0.178	0.0319	0.01
7B_SNP vs. 7B_silicoDArT	0.145	0.0210	0.01
1R_SNP vs. 1R_silicoDArT	0.335	0.1119	0.01
2R_SNP vs. 2R_silicoDArT	0.286	0.0818	0.01
3R_SNP vs. 3R_silicoDArT	0.387	0.1500	0.01
4R_SNP vs. 4R_silicoDArT	0.074	0.0055	0.01
5R_SNP vs. 5R_silicoDArT	0.041	0.0017	0.09
6R_SNP vs. 6R_silicoDArT	0.075	0.0057	0.01
7R_SNP vs. 7R_silicoDArT	0.114	0.0131	0.01

**Table 2:** The Mantel’s test correlation coefficients (*r*) evaluated between genetic distance matrices calculated based on SNPs (above diagonal) and silicoDArTs (below diagonal) for individual chromosomes and the whole pool of markers of the given molecular type.

	Total	1A	2A	3A	4A	5A	6A	7A	1B	2B	3B	4B	5B	6B	7B	1R	2R	3R	4R	5R	6R	7R
Total		0.624	0.613	0.701	0.720	0.717	0.713	0.689	0.644	0.755	0.743	0.432	0.795	0.388	0.608	0.529	0.570	0.646	0.663	0.736	0.717	0.587
1A	0.676		0.377	0.462	0.470	0.458	0.460	0.445	0.435	0.496	0.484	0.262	0.489	0.212	0.334	0.309	0.304	0.362	0.363	0.416	0.386	0.329
2A	0.606	0.334		0.413	0.421	0.417	0.419	0.389	0.380	0.470	0.434	0.251	0.474	0.229	0.345	0.487	0.442	0.344	0.363	0.401	0.375	0.295
3A	0.634	0.399	0.404		0.487	0.488	0.502	0.538	0.467	0.531	0.528	0.275	0.544	0.266	0.435	0.311	0.388	0.479	0.452	0.468	0.432	0.346
4A	0.602	0.477	0.331	0.373		0.520	0.538	0.486	0.451	0.547	0.516	0.321	0.581	0.262	0.455	0.329	0.392	0.412	0.482	0.512	0.481	0.433
5A	0.763	0.555	0.441	0.456	0.486		0.509	0.457	0.423	0.546	0.530	0.308	0.582	0.272	0.449	0.340	0.398	0.432	0.458	0.605	0.481	0.336
6A	0.460	0.313	0.325	0.327	0.355	0.389		0.476	0.445	0.550	0.537	0.294	0.560	0.301	0.389	0.403	0.364	0.421	0.401	0.500	0.555	0.344
7A	0.797	0.620	0.430	0.466	0.513	0.647	0.414		0.433	0.495	0.475	0.290	0.493	0.224	0.376	0.348	0.300	0.387	0.532	0.438	0.422	0.389
1B	0.780	0.562	0.411	0.456	0.454	0.626	0.329	0.599		0.471	0.447	0.256	0.498	0.253	0.435	0.575	0.344	0.373	0.389	0.424	0.403	0.320
2B	0.832	0.569	0.466	0.441	0.481	0.664	0.347	0.671	0.709		0.553	0.314	0.579	0.262	0.437	0.368	0.561	0.427	0.472	0.529	0.485	0.368
3B	0.810	0.487	0.497	0.598	0.417	0.588	0.346	0.617	0.605	0.628		0.311	0.572	0.266	0.420	0.355	0.368	0.577	0.445	0.515	0.501	0.352
4B	0.572	0.402	0.347	0.378	0.338	0.459	0.335	0.469	0.438	0.502	0.487		0.334	0.175	0.234	0.255	0.232	0.273	0.285	0.277	0.261	0.279
5B	0.838	0.571	0.453	0.497	0.510	0.704	0.411	0.675	0.700	0.718	0.689	0.516		0.288	0.626	0.370	0.411	0.451	0.478	0.642	0.552	0.400
6B	0.675	0.419	0.375	0.485	0.427	0.546	0.358	0.517	0.537	0.577	0.542	0.405	0.613		0.229	0.179	0.190	0.233	0.245	0.261	0.324	0.187
7B	0.699	0.470	0.386	0.434	0.487	0.577	0.306	0.612	0.525	0.564	0.521	0.357	0.563	0.467		0.232	0.328	0.348	0.428	0.412	0.374	0.319
1R	0.672	0.524	0.317	0.357	0.299	0.501	0.276	0.511	0.711	0.567	0.508	0.368	0.563	0.381	0.383		0.286	0.363	0.342	0.342	0.298	0.238
2R	0.690	0.392	0.449	0.381	0.409	0.445	0.221	0.447	0.574	0.699	0.465	0.321	0.513	0.464	0.445	0.443		0.358	0.357	0.398	0.340	0.240
3R	0.505	0.176	0.317	0.475	0.136	0.253	0.115	0.292	0.312	0.294	0.666	0.244	0.370	0.276	0.251	0.325	0.298		0.408	0.407	0.386	0.339
4R	0.791	0.527	0.444	0.502	0.452	0.590	0.298	0.643	0.569	0.594	0.660	0.489	0.634	0.480	0.532	0.522	0.446	0.492		0.410	0.390	0.322
5R	0.713	0.477	0.402	0.401	0.467	0.700	0.331	0.593	0.595	0.649	0.517	0.460	0.732	0.515	0.501	0.445	0.449	0.151	0.460		0.519	0.295
6R	0.758	0.530	0.436	0.440	0.452	0.448	0.626	0.659	0.591	0.642	0.590	0.458	0.671	0.586	0.516	0.478	0.415	0.242	0.547	0.595		0.307
7R	0.760	0.582	0.455	0.450	0.487	0.565	0.332	0.651	0.555	0.613	0.593	0.462	0.636	0.479	0.559	0.423	0.440	0.338	0.602	0.493	0.589	

**Table 3:** Structure results for total SNP markers and, according to the assignment, the particular triticale chromosomes, together with the analysis of molecular variance (AMOVA) of the genetic variation between accessions forming groups evaluated by Bayesian analysis.

	Total SNP	1A	2A	3A	4A	5A	6A	7A	1B	2B	3B	4B	5B	6B	7B	1R	2R	3R	4R	5R	6R	7R
Structure analysis																						
K	4	3	2	2	4	3	2	2	2	3	3	2	2	2	2	2	3	3	2	2	2	2
Delta K	2460.2	811.3	2236.7	1625.3	530.4	508.3	1058.7	1261.5	2599.1	644.3	665.7	3429.0	1042.5	1443.3	555.9	910.9	525.4	1994.2	3577.7	2873.3	2881.7	6663.0
No. of accessions in Pop1	276	151	218	241	157	156	120	216	274	110	120	203	216	171	298	301	128	125	249	306	321	168
No. of accessions in Pop2	76	213	228	205	108	191	326	230	172	268	193	243	230	275	148	145	277	259	197	140	125	278
No. of accessions in Pop3	31	82			109	99				68	133						41	62				
No. of accessions in Pop4	63				72																	
Analysis of molecular variance																						
*Phi_PT_*	0.139	0.196	0.316	0.188	0.191	0.181	0.175	0.268	0.231	0.201	0.187	0.305	0.113	0.239	0.015	0.259	0.224	0.159	0.004	0.279	0.347	0.413

*Phi_PT_*—genetic differentiation observed among populations. Ranges from 0 to 0.05, 0.05 to 0.15, 0.15 to 0.25, and >0.25 indicate little, moderate, large, and great genetic differentiation, respectively.

**Table 4:** Pairwise *Phi_PT_* genetic distances between populations with K > 2 for selected triticale chromosomes.

	Total SNP			1A			4A			5A		2B		3B		2R		3R	
Pops	Pop1	Pop2	Pop3	Pop1	Pop2	Pop3	Pop1	Pop2	Pop3	Pop1	Pop2	Pop1	Pop2	Pop1	Pop2	Pop1	Pop2	Pop1	Pop2
Pop1	0			0			0			0		0		0		0		0	
Pop2	0.150	0		0.164	0		0.166	0		0.179	0	0.165	0	0.158	0	0.170	0	0.205	0
Pop3	0.237	0.169	0	0.204	0.243	0	0.211	0.161	0	0.179	0.188	0.253	0.232	0.215	0.193	0.263	0.351	0.225	0.015
Pop4	0.133	0.119	0.163				0.204	0.207	0.226										

**Table 5:** Distribution and linkage disequilibrium of SNP markers on whole genome (WG) and particular chromosomes of triticale.

	WG	1A	2A	3A	4A	5A	6A	7A	1B	2B	3B	4B	5B	6B	7B	1R	2R	3R	4R	5R	6R	7R
Number of markers	4640	95	214	183	139	179	151	236	174	242	216	93	215	152	166	224	300	243	272	412	416	318
Mean distance (Mb)	4.24	6.28	3.64	4.14	5.33	3.95	4.10	3.10	4.13	3.30	5.26	7.32	3.31	4.75	4.45	4.59	3.15	5.60	3.33	2.68	2.53	4.12
Significant LD (%)	35.96	11.6	38.08	40.09	25.34	28.09	32.95	55.15	47.37	29.96	43.10	23.41	35.63	32.00	47.07	30.76	33.54	34.12	31.75	35.11	47.80	49.53
LD (*r*^2^)	0.122	0.031	0.183	0.106	0.078	0.068	0.093	0.223	0.132	0.091	0.104	0.094	0.088	0.088	0.129	0.079	0.168	0.114	0.096	0.114	0.165	0.228
% *r*^2^ < 0.1	77.9	94.1	69.3	77.6	85.4	85.6	83.4	55.7	68.8	81.5	78.0	84.0	81.4	82.7	64.6	85.5	70.6	81.3	80.3	76.1	65.2	70.4
LD Decay (Mb)	8.86	1.18	18.23	5.79	4.79	4.16	3.21	27.48	13.66	5.05	11.60	12.62	7.15	5.80	17.03	4.02	10.98	11.01	4.77	5.66	16.13	28.36

## Data Availability

The original contributions presented in the study are included in the article/Supplementary Material, further inquiries can be directed to the corresponding authors.

## Supplementary Materials

The following supporting information can be downloaded at: https://www.mdpi.com/article/10.3390/ijms25179568/s1.
