# Supplementary material for: Effect of Chromosomal Localization of NGS-Based Markers on Their Applicability for Analyzing Genetic Variation and Population Structure of Hexaploid Triticale
Source: Int J Mol Sci. 2024 Sep 3;25(17):9568. doi: 10.3390/ijms25179568 (PMC11395606; doi:10.3390/ijms25179568)
Supplement: Supplementary file 1 [file ijms-25-09568-s001.zip › ijms-3121246-supplementary.pdf]

Supplementary materials

Effect of chromosomal localization of NGS-based markers on their applicability for analyzing genetic variation and population structure of hexaploid triticale

Justyna Leśniowska-Nowak[1], Piotr T. Bednarek[2], Karolina Czapla[3], Michał Nowak[1]*, and Agnieszka Niedziela[2,]*

[1] Institute of Plant Genetics, Breeding and Biotechnology, University of Life Sciences in Lublin, 20-950 Lublin, Poland
[2] Plant Breeding and Acclimatization Institute – National Research Institute, Radzików, 05-870 Błonie, Poland
[3] Chair and Department of Biochemistry and Molecular Biology, Medical University of Lublin, 20-093 Lublin, Poland
* Correspondence: a.niedziela@ihar.edu.pl (A.N.); michal.nowak@up.lublin.pl (M.N.)

Content:

Figure S1 Genomic distributions of SNPs and silicoDArTs across 21 triticale chromosomes.

Figure S2 Triticale accession's grouped according to their origin: A) UPGMA dendrogram based on SNP markers, B) UPGMA dendrogram based on silicoDArT markers C) PCoA plot based on SNP markers, D) PCoA plot based on silicoDART markers.

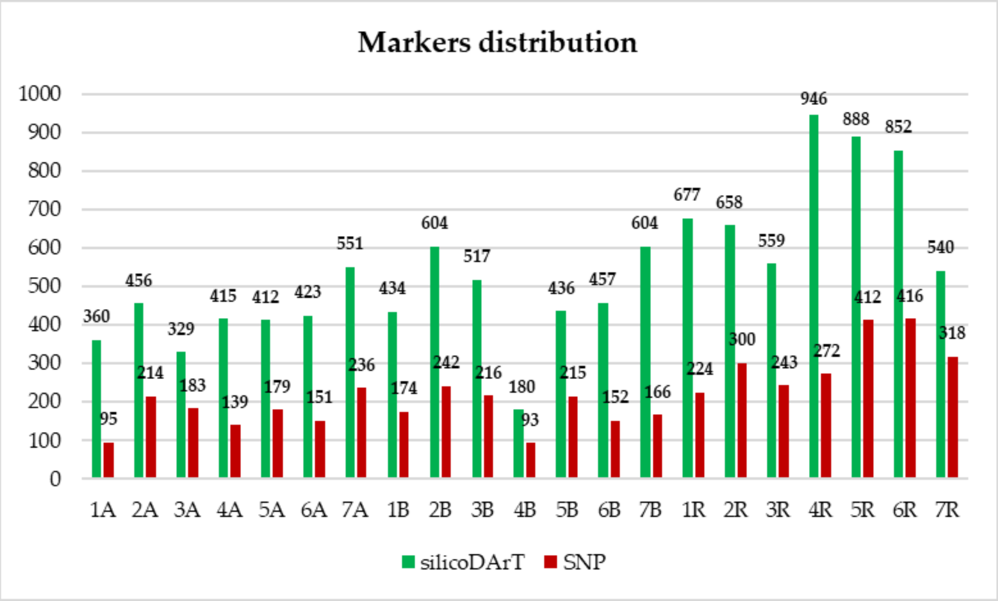

Figure S1 Genomic distributions of SNPs and silicoDArTs across 21 triticale chromosomes.

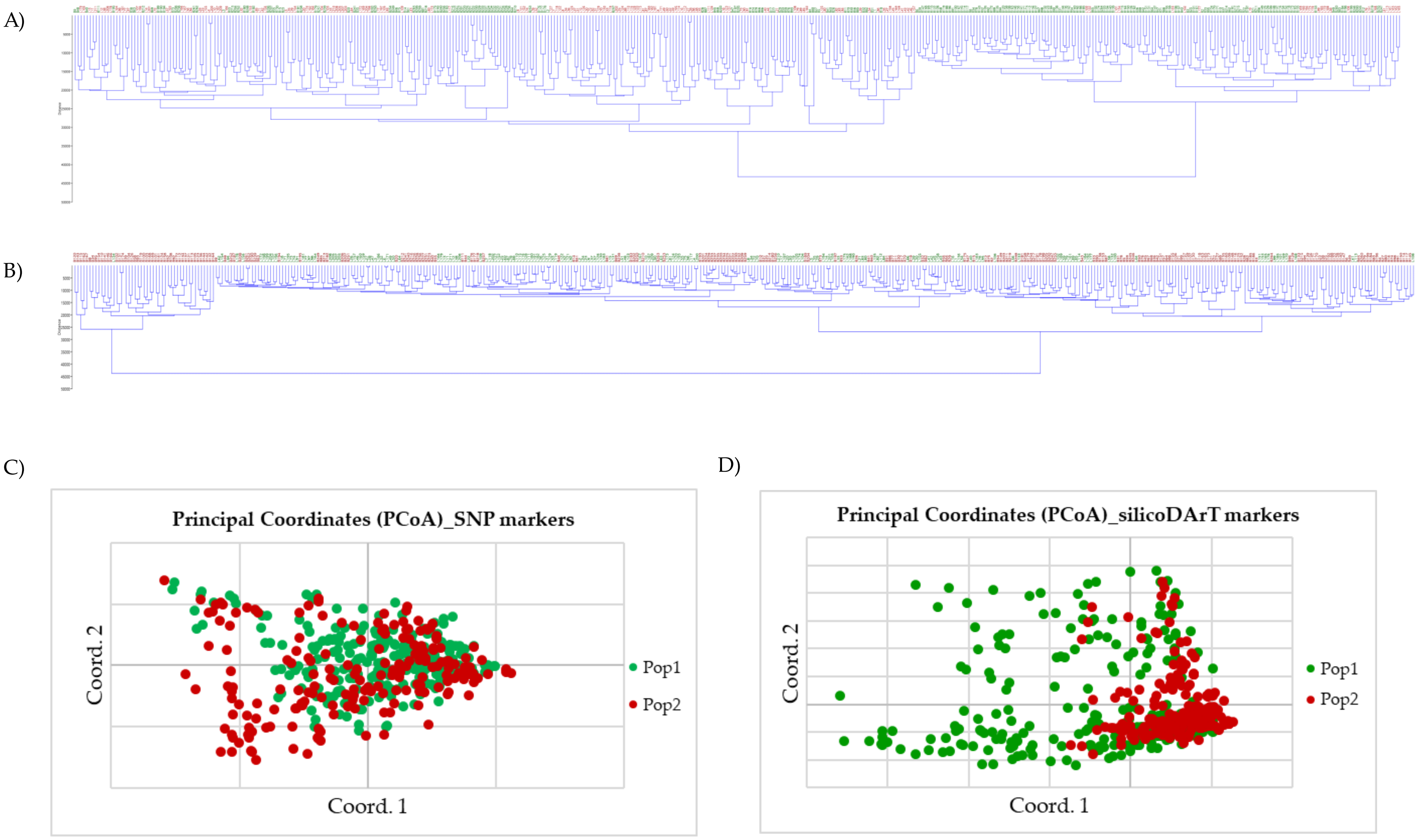

Figure S2 Triticale accession's grouped according to their origin: A) UPGMA dendrogram based on SNP markers, B) UPGMA dendrogram based on silicoDArT markers C) PCoA plot based on SNP markers, , D) PCoA plot based on silicoDART markers.
